# Diagnostic and cost utility of whole exome sequencing in peripheral neuropathy

**DOI:** 10.1002/acn3.409

**Published:** 2017-04-26

**Authors:** Maie Walsh, Katrina M. Bell, Belinda Chong, Emma Creed, Gemma R. Brett, Kate Pope, Natalie P. Thorne, Simon Sadedin, Peter Georgeson, Dean G. Phelan, Timothy Day, Jessica A. Taylor, Adrienne Sexton, Paul J. Lockhart, Lynette Kiers, Michael Fahey, Ivan Macciocca, Clara L. Gaff, Alicia Oshlack, Eppie M. Yiu, Paul A. James, Zornitza Stark, Monique M. Ryan, the Melbourne Genomics Health Alliance

**Affiliations:** ^1^ Murdoch Childrens Research InstituteMelbourneAustralia; ^2^ Royal Melbourne HospitalMelbourneAustralia; ^3^ Melbourne Genomics Health AllianceMelbourneAustralia; ^4^ University of MelbourneMelbourneAustralia; ^5^ Bruce Lefroy CentreMurdoch Childrens Research InstituteParkvilleAustralia; ^6^ Department of PaediatricsThe University of MelbourneMelbourneAustralia; ^7^ Royal Children's HospitalMelbourneAustralia

## Abstract

**Objective:**

To explore the diagnostic utility and cost effectiveness of whole exome sequencing (WES) in a cohort of individuals with peripheral neuropathy.

**Methods:**

Singleton WES was performed in individuals recruited though one pediatric and one adult tertiary center between February 2014 and December 2015. Initial analysis was restricted to a virtual panel of 55 genes associated with peripheral neuropathies. Patients with uninformative results underwent expanded analysis of the WES data. Data on the cost of prior investigations and assessments performed for diagnostic purposes in each patient was collected.

**Results:**

Fifty patients with a peripheral neuropathy were recruited (median age 18 years; range 2–68 years). The median time from initial presentation to study enrollment was 6 years 9 months (range 2 months–62 years), and the average cost of prior investigations and assessments for diagnostic purposes AU$4013 per patient. Eleven individuals received a diagnosis from the virtual panel. Eight individuals received a diagnosis following expanded analysis of the WES data, increasing the overall diagnostic yield to 38%. Two additional individuals were diagnosed with pathogenic copy number variants through SNP microarray.

**Conclusions:**

This study provides evidence that WES has a high diagnostic utility and is cost effective in patients with a peripheral neuropathy. Expanded analysis of WES data significantly improves the diagnostic yield in patients in whom a diagnosis is not found on the initial targeted analysis. This is primarily due to diagnosis of conditions caused by newly discovered genes and the resolution of complex and atypical phenotypes.

## Introduction

Hereditary neuropathies are a group of diverse conditions caused by mutations in more than 60 genes.^1^ Of these, Charcot–Marie–Tooth disease (CMT) is the commonest inherited neuromuscular disorder, affecting 1 in 2500 people.^2^ Neuropathies can also be a feature of other neuromuscular or neurodegenerative conditions and may occur as part of a genetic syndrome or metabolic disorder. A “traditional” diagnostic evaluation produces a definitive genetic diagnosis in around 65% of patients with CMT.^3^, ^4^ Of these, around 45% have chromosome 17p11.12 duplications that include *PMP22* and cause CMT1A.^3^, ^4^, ^5^, ^6^

A number of studies have investigated the utility of next generation sequencing (NGS) approaches in neuropathy patients.^7^, ^8^, ^9^, ^10^, ^11^, ^12^, ^13^ The diagnostic yields of these studies ranged from 19 to 53%. Three employed a NGS‐targeted neuropathy panel.^7^, ^11^, ^13^ Three performed whole exome sequencing (WES) but only analyzed variants from a pre‐selected list of 50–75 genes.^8^, ^9^, ^10^, ^12^ Schabhuttl et al. (2014) employed a staggered approach, initially analyzing variants in neuropathy genes then progressing to gene discovery using linkage analysis.^12^ Recently, Wang et al. (2016) found that incorporating copy number analysis into NGS significantly improved diagnostic efficiency in neuropathy patients, and resulted in a 20% cost savings.^13^

This study aimed to investigate the utility of WES in individuals with presumed genetic peripheral neuropathies and determine strategies to optimize its diagnostic yield. It also explores the health economics of WES in this cohort of patients, and the point in the diagnostic pathway where WES can be most effectively utilized.

## Methods

### Standard protocol approvals and patient consents

This study was conducted between February 2014 and December 2015, as part of the Melbourne Genomics Health Alliance demonstration project (www.melbournegenomics.org.au). It received Human Research Ethics Committee (HREC) approval (13/MH/326). Patients (or their guardians) provided written informed consent after genetic counseling.

Expanded analysis of the exome data was performed with additional consent (the Royal Children's Hospital Ethics Committee, HREC number 28097, version 10).

### Study design and participants

**Figure 1:**
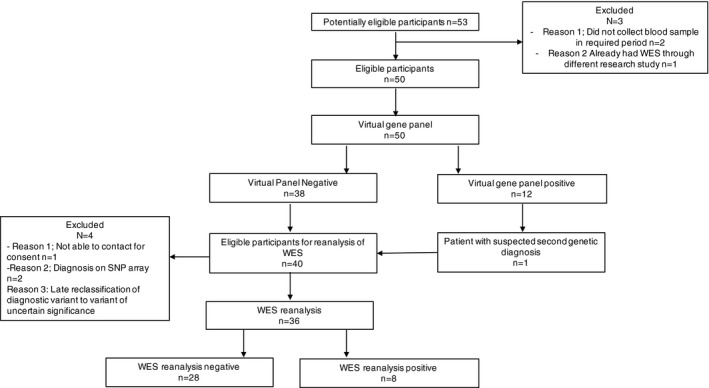
Flow of participants through the study. Abbreviations: WES = Whole exome sequencing.

A cohort of 50 individuals with peripheral neuropathies was prospectively enrolled by a neurologist, or geneticist, and genetic counselor at the Royal Children's Hospital or the Royal Melbourne Hospital, Melbourne Australia. The flow of participants through the study is represented in Figure 1. To be eligible for enrollment, all subjects had to have a neurophysiologically confirmed peripheral neuropathy considered likely to have a monogenic cause. All participants were under ongoing investigation into the cause of their neuropathy in a dedicated clinic. Where suspected clinically, CMT1A caused by *PMP22* duplication was excluded by microarray or Multiplex Ligation‐dependent Probe Amplification (MLPA) prior to study enrollment. Prioritization and exclusion criteria are summarized in Table 1.

**Table 1:** Prioritization and exclusion criteria for entry into the study

Prioritization criteria	Exclusion Criteria
Kindreds with probable dominant or recessive inheritance‐ based on family history and/or known consanguinity‐ were prioritized before isolated sporadic cases Kindreds with probable dominant or recessive inheritance‐ based on family history and/or known consanguinity‐ were prioritized Patients with clinical findings suggestive of an inflammatory neuropathy (such as a raised cerebrospinal fluid protein, conduction block on nerve conduction studies (NCS) and thickened, enhancing nerve roots on MRI) could be included where they had proven refractory to immunosuppressive therapy or treatment for leprosy Patients with chronic idiopathic axonal polyneuropathies (CIAPs) and a family history of adult‐onset distally predominant neuropathy	Patients with sporadic or autosomal dominant neuropathies with typical homogeneous slowing of motor nerve conduction (median motor NCV <38 m/sec) underwent testing for the chromosome 17p duplication involving *PMP22* by microarray or multiplex ligation probe amplification (MLPA) prior to inclusion in the study. Patients with deletions or duplication in that gene were excluded from the study Patients with a consistent phenotype‐ males with distantly predominant weakness, with a typical pattern of split hand involvement (abductor pollicis brevis being more wasted and weaker than the first dorsal interosseus), and intermediate slowing of nerve conduction (25–45 m/sec) and/or patchy changes on NCS, underwent Sanger sequencing of *GJB1* prior to inclusion in the study. Patients with mutations in that gene were excluded from the study Patients with a proven non‐genetic cause were excluded from the study

Exome sequencing, variant detection and variant filtering were performed as previously described (Stark et al. 2016,^14^ Sadedin et al. 2015^15^). The mean coverage obtained was 164 (105‐256).

Variants were classified according to the American College of Medical Genetics and Genomics standards for interpretation.^16^, ^17^ Classifications were discussed in a multidisciplinary meeting by clinical geneticists, neurologists, genetic counselors, molecular geneticists and bioinformaticians. Sanger sequencing to confirm pathogenic and likely pathogenic variants was performed at an independent clinical laboratory. Family segregation studies were performed where necessary to clarify pathogenicity.

**Figure 2:**
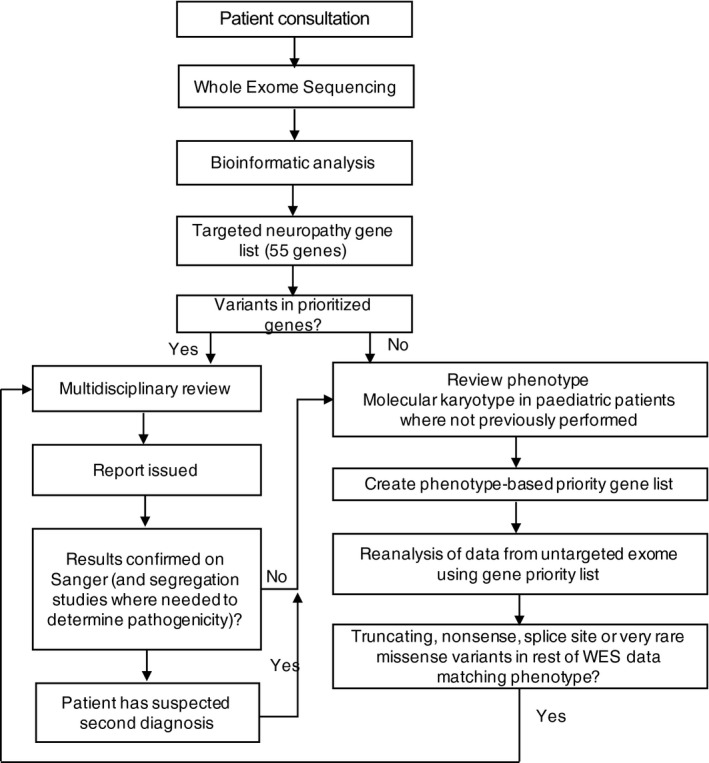
Process for recruitment, sequencing and data analysis. Abbreviations: WES = Whole exome sequencing.

Initial analysis was restricted to variants in a predetermined virtual panel of 55 genes associated with hereditary neuropathies as of 2013 (Table S1 and S2). The mean coverage obtained these genes were 159 (113‐245). This list was generated by a review of online resources such as http://www.musclegenetable.fr and PUBMED. Patients with uninformative results, or a result that only explained part of their phenotype, were re‐phenotyped by a clinical geneticist with input from neurologists. Single nucleotide polymorphism (SNP) microarray testing was undertaken by an accredited clinical laboratory in pediatric patients were not done previously. Re‐analysis was focused on an expanded neuropathy gene list (88 genes, see Table S2, generated in 2015). Additional genes were found from a literature search using the terms: “Charcot–Marie–Tooth disease,” “hereditary sensory neuropathy” and “hereditary motor neuropathy” and a list from Rosser et al. 2013.^1^ Patients with additional features suggestive of an underlying syndrome had customized gene lists generated based on their phenotype. Additional features were defined as neurologic or systemic findings, such as ataxia, spasticity, congenital anomalies or intellectual disability that were suggestive of the patient being affected by an underlying syndrome or different neuromuscular condition rather than a “pure” CMT phenotype. Manual examination of high‐priority candidate genes was undertaken where necessary. Where a diagnosis was not reached following these phenotype‐based prioritization approaches, all truncating, nonsense, splice‐site and very rare conserved variants were reviewed. Only those found in genes associated with a phenotype concordant with that of the patient were assessed for pathogenicity. The process for recruitment, sequencing and data analysis is described in Figure 2. Where needed, RNA studies were performed to confirm the effect of splice site variants.

### Costs

A clinical geneticist reviewed all patient medical records and collated all investigations, procedures and assessments that took place primarily for diagnostic purposes. The costs of investigations and encounters were obtained from hospitals, state government and from testing laboratories. For the pediatric cohort, the first three appointments with any neurologist were considered as being for diagnostic purposes. For the adult cohort the patient's first appointments with a new private neurologist/public neurology service were included, together with all appointments in neurogenetics clinics.

### Scenario analysis

To explore the impact of early implementation of WES, a hypothetical scenario was developed using an approach similar to that of van Nimwegen et al.^18^ The scenario assumed that WES could replace sequencing‐based genetic tests, repeated nerve conduction studies, complex biochemical tests and tissue biopsies. In this scenario the costs of sample preparation and shipping to other laboratories, and theatre and anesthetic costs, were eliminated. Neurology appointments for diagnostic purposes were limited to two per patient.

## Results

### Participant demographics and indications for testing

Fifty‐three individuals were eligible for inclusion; three withdrew from the study. The type of neuropathy and clinical characteristics of the 50 enrolled participants are summarized in Table 2. The median age was 18 (range 2–68 years). Thirteen patients had additional features.

**Table 2:** Characteristics of enrolled patients, and diagnostic rate in subgroups

Characteristic	*n* (%)	Diagnosis achieved by analysis of:	
		Virtual panel *n* (%)	WES *n* (%)
Sex			
Male	33 (66)	5 (15)	11 (33)
Female	17 (34)	6 (35)	8 (47)
Age at enrollment			
0–17 years	23 (46)	4 (17)	7 (15)
18 years or older	27 (54)	7 (26)	12 (44)
Parental consanguinity	5 (10)	0 (0)	3 (60)
Affected first‐degree relative	15 (30)	2 (13)	3 (20)
Neurophysiological phenotype			
Demyelinating sensorimotor neuropathy	9 (18)	3 (33)	4 (44)
Axonal sensorimotor neuropathy	17 (34)	0 (0)	4 (24)
Intermediate sensorimotor neuropathy	10 (20)	4 (40)	5 (50)
Pure motor neuropathy	11 (22)	4 (36)	5 (45)
Pure sensory neuropathy	3 (6)	0 (0)	1 (33)
Additional systemic features	13 (26)	1 (8)	5 (39)
Previous genetic testing			
No prior testing	10 (20)	3 (30)	5 (50)
1‐4 tests	34 (68)	7 (21)	13 (38)
>5 tests	6 (12)	1 (17)	1 (17)

### Diagnostic yield

A diagnosis was achieved in 11 of 50 participants during initial analysis restricted to the virtual panel, giving a diagnostic rate of 22%. The clinical features and pathogenic and likely pathogenic variants found in these individuals are presented in Table S3. One result (0102025) was not confirmed on Sanger sequencing due to the patient not providing a second sample. However, as this was a phenotype‐concordant, previously reported *PMP22* variant of good quality (present in 87 out of 162 reads), this result was considered diagnostic.

Thirty‐eight of for eligible patients were enrolled in expanded analysis of the entire WES data. Thirty‐eight of these remained undiagnosed following the initial targeted analysis and one patient (0202262) was enrolled because she had additional features suggestive of a second genetic condition. Two patients were found to have chromosome 17p12 duplications including *PMP22* on SNP microarray, consistent with CMT1A, prior to expanded WES analysis; these subjects were withdrawn from further investigation.

Seven out of thirty‐six patients achieved a diagnosis following re‐phenotyping and expanded WES analysis (Table S3 and S4). An eighth patient had a homozygous novel intronic variant in the *SBF2* gene (NM_030962.3(*SBF2*):c.620‐9T>A) that required RNA studies to confirm pathogenicity. cDNA derived from control lymphoblast and fibroblast cells demonstrated the expected 399 bp fragment spanning exons 6‐8 of *SBF2*. In comparison, patient lymphoblast and fibroblast cells did not generate any full‐length product, instead a fragment of ~280 bp was observed, consistent with exon 7 skipping. This is predicted to result in a frameshift and premature termination of the protein (NP_112224.1(SBF2):p.Gly207Valfs*16). (Fig. S1).

### Health care utilization and cost data including scenario analysis

The two patients in whom a diagnosis was made on microarray were excluded from the cost analysis. Patients were enrolled in different stages of their diagnostic trajectory with a median of 6 years 9 months (range 2 months–62 years) from clinical diagnosis to date of recruitment.

On average patients had 3.4 (range 1–9) specialty appointments purely for diagnostic purposes. Seventeen patients had additional neurophysiological investigations (most often repeat NCS). Eight patients underwent at least one invasive test, including lumbar puncture or nerve and muscle biopsy.

Forty (80%) of patients had undergone some form of prior genetic testing. In some cases, patients had undergone limited genetic testing because an affected relative had already had the relevant investigations. The average number of genetic tests prior to enrollment was 2 (range 0–6). Five patients had prior testing in the form of a targeted neuropathy gene panel.

In total AU$192,627 was spent on prior investigations and assessments for diagnostic purposes in this cohort, with an average cost of AU$4,013 per patient (SD AU$2,761 range AU$526‐$13,128). Performing WES cost AU$111,894 (AU$2000 for clinical WES and AU$331 for two consultations with a genetic counsellor per patient) resulting in an average cost per diagnosis of AU$16,027. If WES had been performed at an earlier point in the diagnostic trajectory as per the scenario analysis, the average cost per diagnosis would have been AU$12,413 resulting in a total cost saving of 22.5%. A table summarizing the cost data is available in the supplementary information (Table S5).

## Discussion

Inherited neuropathies are ideal candidates for diagnosis by NGS approaches. The place of targeted sequencing panels and WES (with analysis restricted to a virtual gene panel or not) in diagnostic pathways has not been established. In this study, we used a tiered approach beginning with analysis restricted to a virtual panel progressing through to analysis of the rest of the WES data in unsolved cases. Expanded WES analysis increased the diagnostic yield by 73%. Our overall yield of 38% from WES is comparable to other studies, particularly as CMT1A had largely been excluded prior to enrollment.

While the sample size was relatively small, the distribution of genetic sub‐types diagnosed on the virtual panel generally reflected the findings from larger studies. Ten of 11 patients (91%) who achieved a diagnosis from this initial analysis had mutations in one of the five most common causes of genetically diagnosed CMT (*GJB1, MPZ, MFN2, SH3TC2, PMP22*) in larger series (excluding *PMP22* duplications).^3^, ^4^, ^5^, ^6^, ^19^

This study highlights the advantages of applying WES in this cohort. Firstly, with the advent of NGS the number of genes known to be associated with CMT and related conditions is continuing to grow, with a 70% increase between 2009 and 2013.^1^ Unlike targeted NGS panels whereby only pre‐set genes of interest are sequenced, the generation of WES data allows reanalysis as new genes are linked to the patient phenotype. Three of the nine patients who received a diagnosis from the expanded analysis of their WES data in this study had mutations in genes discovered after the contents of the virtual neuropathy panel were defined in 2013: *BICD2*,* PDZD7* and *KIF1A*.^20^, ^21^, ^22^, ^23^

Another group benefitting from expanded WES data analysis was those patients with complex phenotypes. The diagnostic rate in this group following virtual panel analysis was 1/13 (7.7%); this increased to 5/13 (38%) after expanded WES analysis. The presence of additional features may suggest that the neuropathy is part of an underlying syndrome or the possibility of multiple pathologies, raising a diagnostic test selection dilemma. An example was a 4‐year old girl (0202262) with a severe early‐onset neuropathy, thyroid agenesis, and post‐axial polydactyly. Despite the presence of additional features, she was found to have a well‐established pathogenic missense variant in *PMP22,* consistent with Dejerine–Sottas disease. Her other clinical findings remain unexplained. In contrast, we identified a previously reported *de novo* variant in a highly conserved amino acid in *KIF1A* 22 in a 3 year‐old boy (0202254) who presented with a sensory axonal neuropathy, global developmental delay, optic atrophy and cerebellar ataxia; this single pathogenic variant reconciled his entire phenotype. Another patient in this cohort is a 68 year‐old woman (0102018) with bilateral sensorineural hearing loss with onset at 6 years and a progressive axonal neuropathy diagnosed at age 30, who was found to have a previously reported truncating mutation and a canonical splice site mutation in *PDZD7*. This gene has recently been associated with childhood onset autosomal recessive, non‐syndromic hearing loss in five families.^23^, ^24^ As she is the first adult described with biallelic mutations in *PDZD7,* it is unclear whether her adult‐onset neuropathy is caused by *PDZD7* or whether she has a second undiagnosed condition.

Patients with atypical presentations are another group benefitting from the expanded WES analysis. An example of this is patient (0202259) who presented with an axonal neuropathy and a history of mild speech delay. He was found to have novel compound heterozygous mutations in *SLC12A6,* consistent with a diagnosis of Hereditary Motor and Sensory Neuropathy with Agenesis of the Corpus Callosum (Andermann syndrome). This diagnosis was not suspected clinically as he appeared to have an essentially isolated neuropathy and had a structurally normal brain on MRI. However, agenesis of the corpus callosum is only present in around 30% of cases of Andermann syndrome.^25^ In addition, while Andermann syndrome is traditionally associated with a severe neurodegenerative phenotype, there are reports of milder phenotypes associated with missense mutations including one patient who initially presented with features of a pure neuropathy.^26^, ^27^ Two adult patients with neuropathy were diagnosed with different neuromuscular conditions following expanded analysis, including a previously reported *REEP1* mutation associated with spastic paraplegia (0102015) and a patient with suspected CMT5, who was found to have compound heterozygous mutations in *SACS* consistent with spastic ataxia of the Charlevoix–Saguenay type (0102007).

Our experience underscores the importance of copy number analysis in the work‐up of patients with genetic neuropathies. Of the two patients who were diagnosed with CMT1A using SNP microarray following an uninformative result on the virtual panel, one had an affected brother who had previously had a negative result on *PMP22* MLPA. The brother was subsequently confirmed to have *PMP22* duplication on SNP microarray.

SNP microarray also adds value in delineating regions of homozygosity which can be used to identify high‐priority candidate genes.^28^ One subject (0202259) had an early onset demyelinating neuropathy with myelin outfoldings on nerve biopsy, a highly specific finding. She was one of two children to her consanguineous parents; no other family members were affected. Her SNP microarray showed multiple regions of homozygosity, accounting for around 2% of the genome. Her phenotype was consistent with CMT4 and the only gene within a region of homozygosity consistent with this was *SBF2*. No variants in *SBF2* were found on initial automated bioinformatic analysis, or on a previously performed targeted neuropathy gene panel. On manual examination of the patient's WES data we found a homozygous novel intronic sequence variant (NM_030962.3(*SBF2*):c.620‐9T>A) affecting a highly conserved base; subsequent RNA studies confirmed that this resulted in a frameshift and premature stop codon, p.(Gly207Valfs*16).

The average cost per patient for prior diagnostic work‐up in this cohort was substantial, but interestingly less than that reported in three other studies looking at patients with neurological disorders which reported average costs of US$19,000^29^, ^30^ and €12,475.^18^ This may reflect the fact that several of the patients in this study were beginning their diagnostic trajectory, or the constraints on expenditure within the publically funded healthcare system in Australia. Regardless, the scenario analysis demonstrates a considerable cost saving (~22.5%) associated with introducing WES earlier in the diagnostic trajectory in this cohort. This finding is in keeping with the literature on WES in other disease cohorts including subjects with epilepsy and intellectual disability.^29^, ^30^, ^31^

This study was limited by a small sample size. Studies investigating the impact of a genetic diagnosis on management, reproductive choices and psychosocial impact for patients with neuropathies and their families are needed. The cost of health utilization is likely to be an underestimate of the amount spent, as many of the patients have been investigated across several hospitals over many years and it is likely that not all investigations and appointments were captured. The cost analysis also does not take into account additional costs associated with the ongoing investigation of patients who did not achieve a diagnosis from WES, or costs associated with a new genetic diagnosis such as screening for additional complications or investigation of family members. There were no incidental findings in this study, however the higher risk of incidental findings in WES compared to single gene or targeted panel analysis is an important ethical issue to consider in test selection and pre‐test counseling.

This study demonstrates the clinical diagnostic utility of WES in patients with a suspected genetic neuropathy. While the use of predetermined virtual panels undoubtedly facilitates efficient, phenotype‐driven WES data analysis in large numbers of patients, expanded analysis of unsolved cases allows identification of conditions caused by newly discovered genes and diagnosis of patients with atypical and/or complex clinical presentations. WES could be considered as a first‐line investigation for patients presenting a neuropathy, in whom there is a strong suspicion of genetic etiology but definitive diagnosis cannot be established through baseline tests such as nerve conduction studies and microarray. Where a mutation in one of the common neuropathy genes is considered likely and there are significant cost savings, testing of these prior to WES may be considered. The early use of WES maximizes the diagnostic yield while reducing the time to diagnosis and the financial and psychological burdens associated with prolonged investigation.

## Author Contributions

MW and PAJ performed study concept and design, analysis and of interpretation of data, drafting and revising of the manuscript, acquisition of data and statistical analysis. KMB, BC, DPG, TD, EMY and LK performed analysis and of interpretation of data, acquisition of data, revising of the manuscript. EC and GRB performed patient recruitment and consent, acquisition of data, Analysis and of interpretation of data and revising of the manuscript. KP and MF performed acquisition of data and revising of the manuscript. NPT, SS, PG, AS and JAT performed analysis and of interpretation of data, revising of the manuscript. PJL, IM and CLG performed study concept and design and revising manuscript. AO performed analysis and of interpretation of data, research supervisor, revising of the manuscript. ZS performed study design and concept, drafting and revising of the manuscript, research supervisor, drafting and revising of the manuscript and statistical analysis. MMR performed study design and concept, study supervisor, analysis and of interpretation of data and recruitment, drafting and revising of the manuscript.

## Conflicts of Interest

The authors have no conflicts of interest to declare.

## Supporting information


**Table S1 and S2**. Genes and associated neuropathy phenotypes used for the initial restricted analysis (2013) and additional genes used in the expanded neuropathy gene list used for the reanalysis (2015).

**Table S3 and S4**. Phenotype and gene mutations in patients who received a genetic diagnosis following WES analysis restricted to virtual gene panel, and phenotype and gene mutations in patients who received a genetic diagnosis following expanded analysis of WES data.

**Table S5** Summary of Cost Data.

**Figure S1**. Analysis of alternative splicing of SBF2 in patient lymphoblast and fibroblast cell lines.
