# Supplementary material for: Diagnostic and cost utility of whole exome sequencing in peripheral neuropathy
Source: Ann Clin Transl Neurol. 2017 Apr 26;4(5):318–25. doi: 10.1002/acn3.409 (PMC5420808; doi:10.1002/acn3.409)
Supplement: Supplementary file 1 — Table S1 and S2. Genes and associated neuropathy phenotypes used for the initial restricted analysis (2013) and additional genes used in the expanded neuropathy gene list used for the reanalysis (2015). [file ACN3-4-318-s001.docx]

**Table S1 Genes and associated neuropathy phenotypes used for the initial restricted analysis (2013)**

| **Gene** | **Neuropathy Phenotype (OMIM number)** |
| --- | --- |
| *AARS* | CMT2N (613287) |
| *ATL1* | HSN1D (613708) |
| *ATP7A* | X-linked Distal SMA (300489) |
| *BSCL2* | HMN5A (606158) |
| *CCT5* | AR HSN with spastic paraplegia (256840) |
| *CTDP1* | Congenital cataracts, facial dysmorphism, and neuropathy (604168) |
| *DCTN1* | Distal HMN7B (607641) |
| *DHTKD1* | CMT2Q (615025) |
| *DNM2* | CMT2M and CMTDIB (606482) |
| *EGR2* | CMT1D (607678), Dejerine-Sottas disease (145900), Congenital hypomyelinating neuropathy (605253) CMT4E |
| *FAM134B* | HSAN2B (613115) |
| *FBLN5* | Hereditary neuropathy with or without age-related macular degeneration (604580) |
| *FGD4* | CMT4H (609311) |
| *FIG4* | CMT4J (611228) |
| *GAN* | Giant axonal neuropathy-1 (256850) |
| *GARS* | CMT2D (601472), HMN5A (600794) |
| *GDAP1* | CMT2K (607831), Axonal CMT with vocal cord paresis (607706), AR intermediate CMTA (608340), CMT4A (214400) |
| *GJB1* | CMTX1 (302800) |
| *HARS* | CMT2W (616625) |
| *HINT1* | AR neuromyotonia and axonal neuropathy (137200) |
| *HK1* | CMT4G (605285) |
| *HSPB1* | CMT2F (606595), HMN2B (608634) |
| *HSPB3* | HMN2C (613376) |
| *HSPB8* | CMT2L (608673), HMN2A (158590) |
| *IGHMBP2* | CMT2S(616155), HMN6 (604320), DSMA1 |
| *IKBKAP* | Familial dysautonomia (223900) |
| *KARS* | Intermediate CMT type 1B |
| *KIF5A* | Spastic paraplegia with or without peripheral neuropathy (604187) |
| *LITAF* | CMT1C (601098) |
| *LMNA* | CMT2B1 (605588) |
| *LRSAM1* | CMT2P (614436) |
| *MARS* | CMT2U (616280) |
| *MED25* | CMT2B2 (605589) |
| *MFN2* | CMT2A2 (609260), CMT6A (601152) |
| *MPZ* | CMT1B ( 118200), CMT2I ( 607677), CMT2J (607336), CMT intermediate D (607791), Dejerine-Sottas disease (145900), |
| *MTMR2* | CMT4B1 (601382) |
| *NDRG1* | CMT4D (601455) |
| *NEFL* | CMT1F (607734), CMT2E (607784) |
| *NGF* | HSAN5 (608654) |
| *NTRK1* | Congenital insensitivity to pain with anhidrosis (256800) |
| *PLA2G6* | Infantile neuroaxonal dystrophy 1 (256600) |
| *PLEKHG5* | CMT intermediate C (613376), Distal SMA (611067) |
| *PMP22* | CMT1A (118220), CMT1E(118300), HNPP(162500), Dejerine-Sottas disease (145900), Inflammatory demyelinating polyneuropathy (139393) |
| *PRPS1* | CMTX5 (311070) |
| *PRX* | CMT4F (614895) |
| *RAB7A* | CMT2B (608882) |
| *SBF1* | CMT4B3 (615284) |
| *SBF2* | CMT4B2 (604563) |
| *SH3TC2* | CMT4C (601596), Mild mononeuropathy of the median nerve (613353) |
| *SPTLC1* | HSAN1A (162400) |
| *SPTLC2* | HSAN1C (613640) |
| *TFG* | Okinawa hereditary motor and sensory neuropathy (604484), AR spastic paraplegia-57 (615658) |
| *TRPV4* | CMT2C (606071) |
| *WNK1* | HSAN2A (201300) |
| *YARS* | Type C AD intermediate CMT (608323) |

Abbreviations: CMT- Charcot Marie Tooth, SMA – spinal muscular atrophy, HMN- Hereditary motor neuropathy, HSAN- Hereditary sensory and autonomic neuropathy, AD- Autosomal dominant, AR-Autosomal recessive.

**Table S2 Additional genes used in the expanded neuropathy gene list used for the reanalysis (2015)**

| Gene | Neuropathy Phenotype (OMIM number) |
| --- | --- |
| *MT-ATP6* | Neuropathy, ataxia and retinitis Pigmentosa (551500), Mitochondrial axonal CMT |
| *TRIM2* | CMT2R |
| *AIFM1* | Cowchock syndrome (310490) |
| *PDK3* | CMTX6 (300905) |
| *IFN2* | AD intermediate CMTE with focal segmental glomerulonephritis (614455) |
| *GNB4* | AD intermediate CMTF (615185) |
| *SETX* | HMN with pyramidal features (602433), AR Spinocerebellar ataxia 1 (606002) |
| *DNAJB2* | CMT2T (616233), AR Distal SMA-5 (614881) |
| *REEP1* | HMN5B (614751), AD Spastic paraplegia- 31 (610250) |
| *SLC5A7* | HMN7A (158580) |
| *BICD2* | AD SMA-2 (615290) |
| *DYNC1H1* | CMT2O (614228), AD SMA-1 (158600) |
| *MYH14* | Peripheral neuropathy, myopathy, hoarseness and hearing loss (614369) |
| *DNMT1* | HSN1E (614116) |
| *KIF1A* | HSN2C (614213), AR Spastic paraplegia- 30 (610357) |
| *SCN9A* | HSAN2D (243000), Small fiber neuropathy (133020) |
| *PRNP* | Prion disease associated with diarrhoea and autonomic neuropathy |
| *IGHMBP2* | CMT2S (616155), HMN5I (604320) |
| *COX6A1* | AR Intermediate CMTD (616039) |
| *VCP* | CMT2Y (616687) |
| *HADHB* | Autosomal recessive axonal CMT |
| *ATL3* | HSNIF (615632) |
| *DCAF8* | AD giant axonal neuropathy (610100) |
| *VRK1* | Complex motor and sensory axonal neuropathy with microcephaly |

Abbreviations: CMT- Charcot Marie Tooth, HMN- Hereditary motor neuropathy, HSAN- Hereditary sensory and autonomic neuropathy, AD- Autosomal dominant, AR-Autosomal recessive, SMA-Spinal muscular atrophy, HSN- Hereditary sensory neuropathy. These additional genes were found from a literature search using the search terms: “Charcot–Marie–Tooth disease”, “hereditary sensory neuropathy” and “hereditary motor neuropathy” and a list from Rosser et al. 2013.^1^
