# Supplementary material for: Diagnostic and cost utility of whole exome sequencing in peripheral neuropathy
Source: Ann Clin Transl Neurol. 2017 Apr 26;4(5):318–25. doi: 10.1002/acn3.409 (PMC5420808; doi:10.1002/acn3.409)
Supplement: Supplementary file 2 — Table S3 and S4. Phenotype and gene mutations in patients who received a genetic diagnosis following WES analysis restricted to virtual gene panel, and phenotype and gene mutations in patients who received a genetic diagnosis following expanded analysis of WES data. [file ACN3-4-318-s002.docx]

**Table S3. Phenotypic characteristics and gene mutations in patients who received a genetic diagnosis following WES analysis restricted to virtual gene panel**

| **Study no, age,**  **Sex** | **Phenotype**  **(HPO term)** | **Diagnosis**  **(OMIM no)** | **Gene** | **Mutation** | **Evidence for pathogenicity** |
| --- | --- | --- | --- | --- | --- |
| 0202260, 13,  M | [HP:0007002] Motor axonal neuropathy, [HP:0001763] Pes planus | CMT2K  (607831) | *GDAP1* | HTZ  NM_018972.2, NP_061845.2, c.358C>T, p.(Arg120Trp) | Absent in population databases  Predicted to be pathogenic by in-silico analysis*  Functional domain  Previously reported as pathogenic by Ammar et al 2005, Zimon et al 2001 and Claramunt et al 2005 |
| 0202264,  18,  F | [HP:0007108] Demyelinating peripheral neuropathy  [HP:0002650] Scoliosis | CMT4C  (601596) | *SH3TC2* | HMZ  NM_024577, NP_078853.2  c.2860C>T, p.(Arg954*) | Truncating variant in a gene where loss of function mutations are a known disease mechanism.  Previously reported as pathogenic by Lupski et al 2010; Houlden at al 2009; Gosselin et al 2008. |
| 0202271,  7,  F | [HP:0007002] Motor axonal neuropathy | CMTX1 (302800) | *GJB1* | HTZ  NM_000166, NP_000157.1  c.643C>T, p.(Arg215Trp) | Absent in population databases  Predicted to be pathogenic by in-silico analysis  Previously reported as pathogenic with functional data by Fairweather et al 1994 and Castro et al 1999  Segregates with phenotype -variant detected in affected mother and half-brother. Not detected in unaffected father and half-sister |
| 0202272,  11,  M, | [HP:0007108] Demyelinating peripheral neuropathy  [HP:0002650] Scoliosis [HP:0006466] Ankle contractures | CMT4C  (601596) | *SH3TC2* | HTZ  NM_024577, NP_078853.2  c.3425_3435del, p.(Tyr1142Phefs*38)  HTZ  NM_024577, NP_078853.2  c.2860C>T,Arg954* | Absent in population databases  Predicted to be pathogenic by in-silico analysis  Truncating variant in a gene where loss-of-function mutations are a known disease mechanism.  Truncating variant in a gene where loss-of-function mutations are a known disease mechanism.  Previously reported as pathogenic by Lupski et al 2010; Houlden at al 2009; Gosselin et al 2008. |
| 0102009,  37,  F, | [HP:0007327] Mixed demyelinating and axonal polyneuropathy | CMTX1 (302800) | *GJB1* | HTZ  NM_000166.5, NP_000157.1  c.94A>G, p.(Arg32Gly) | Not present in population databases  Predicted to be pathogenic by in-silico analysis  Located in functional domain  Previously reported as pathogenic by Keckarevic MP et al 2013 and shown to segregate with disease by Mioltenberger-Miltenyi G et al 2009 |
| 0102010,  35,  F | [HP:0007327] Mixed demyelinating and axonal polyneuropathy | CMTX1 (302800) | *GJB1* | HTZ  NM_000166.5, NP_000157.1  c.65G>A, p.(Arg22Gln) | Not present in population databases  Predicted to be pathogenic by in-silico analysis  Located in functional domain  Previously reported as pathogenic including supportive functional data by Matsuyama et al. 2001 and shown to segregate with disease by Silander et al. 1997 |
| 0102025,  18,  M | [HP:0007108] Demyelinating peripheral neuropathy | HNPP  (162500) | *PMP22* | HTZ  NM_153322, NP_696997.1  c.372G>A, p.(W124X) | Truncating variant in a gene where loss-of-function mutations are a known disease mechanism.  Predicted to be pathogenic by in-silico analysis  Previously reported as pathogenic by Pareyson et al.1996 |
| 0102008,  55,  M | [HP:0007327] Mixed demyelinating and axonal polyneuropathy | CMT 2A2  (609260) | *MFN2* | HTZ  NM_1127660.1, NP_001121132.1,  c.691T>C, p.(Ser231Pro) | Absent in population databases  Predicted to be pathogenic by in-silico analysis  Located in functional domain/mutation hot spot  Patient’s phenotype specific for gene |
| 0102001,  55,  F | [HP:0007327] Mixed demyelinating and axonal polyneuropathy | CMT4C  (601596) | *SH3TC2* | HMZ  NM_024577, NP_078853.2  c.335C>T, p.(Thr112Ile) | Present at very low frequency in population databases (1 in 121356) in heterozygous state only  Predicted to be pathogenic by in-silico analysis  Detected in trans with another variant  Phenotype regarded as specific for gene |
| 0102002,  58,  M, | [HP:0007002] Motor axonal neuropathy | SPG3A  (182600) | *ATL1* | HTZ  NM_015915.4, NP_056999.2  c.650G>A, p.(Arg217Gln) | Absent in population databases  Predicted to be pathogenic by in-silico analysis  Located in functional domain  Previously reported as pathogenic with segregation data by Muglia M. et al 2002 |
| 0202262,  4,  F | [HP:0007002] Severe motor neuropathy, [HP:0002650] Scoliosis, [HP:0000486] Strabismus, [HP:0100259] Post-axial polydactyly, [HP:0001385] Hip dysplasia, [HP:0003396] Syringomyelia, [HP:0000851] Congenital hypothyroidism | DSD  (145900) | *PMP22* | HTZ  NM_153322, NP_696997.1  c.215C>T, p.(Ser72Leu) | Absent in population databases  Predicted to be pathogenic by in-silico analysis  Previously reported as pathogenic by Roa at al 1993; Lonasescu et al 1996; Marques et al 1998 |

Abbreviations: HPO = Human Phenotype Ontology; OMIM = Online Mendelian Inheritance in Man; AD = autosomal dominant; AR = autosomal recessive; XL = X-linked recessive; CMT = Charcot-Marie-Tooth disease; SMA = spinal muscular atrophy; HMN = hereditary motor neuropathy; HSAN-hereditary sensory and autonomic neuropathy; DSD- Dejerine-Sottas disease; HNPP = hereditary neuropathy with liability to pressure palsies; SPG = Spastic paraplegia;

*In-silico prediction tools used Mutationtaster.org (Schwarz JM et al. 2014), Polyphen at http://genetics.bwh.harvard.edu/pph2/ (Adzhubei IA 2010 et al) and <http://sift.jcvi.org> (Kumar P et al 2009),

**Table S4. Phenotypic characteristics and gene mutations in patients who received a genetic diagnosis following expanded analysis of WES data**

| **Study no, age,**  **Sex** | **Phenotype** | **Diagnosis**  **(OMIM no)** | **Gene** | **Mutation** | **Evidence for pathogenicity** |
| --- | --- | --- | --- | --- | --- |
| 0202252,  17,  M | [HP:0007002] Motor axonal neuropathy, [HP:0001385] Hip dysplasia, [HP:0001848] Calcaneovalgus deformity, [HP:0000563] Keratoconus, [HP:0001382] Joint hypermobility | SMA, lower extremity-predominant (615290) | *BICD2* | HTZ  NM_015250.3, NP_001003800.1, c.1617_1618delinsGA, p.(His539_His540delinsGlnAsn) | Absent in population databases  Predicted to be pathogenic by in-silico analysis*  Confirmed *de novo* by segregation studies |
| 0202253,  6,  M | [HP:0003477] Peripheral axonal neuropathy, [HP:0006466] Ankle contractures, [HP:0000750] Delayed speech and language development | Agenesis of the corpus callosum with peripheral neuropathy  (218000) | *SLC12A6* | HTZ  NM_133647.1, NP_ 598408.1  c.3041_3042delAG, p.(Glu1014Glyfs*19)  HTZ  NM_133647.1, NP_ 598408.1  c.1476_1478delCTT, p.(Phe493del) | Absent in population databases  Truncating variant in a gene where loss-of-function mutations are a known disease mechanism.  Predicted to be pathogenic by in-silico analysis  Absent in population databases  Predicted to be pathogenic by in-silico analysis  Located in functional domain  Proven to be in trans with other pathogenic variant on segregation analysis |
| 0202254,  3,  M | [HP:0000763] Sensory neuropathy, [HP:0000648], Optic atrophy, [HP:0000964], Eczema, [HP:0001263] Global developmental delay, [HP:0001257] Spasticity | Autosomal dominant mental retardation 9 (614255) | *KIF1A* | HTZ  NM_001244008, NP_ 001230937.1, c.946C>T, p.(316Arg>Trp) | Absent in population databases  Predicted to be pathogenic by in-silico analysis  Located in functional domain  Confirmed *de novo* by segregation studies  Previously reported as pathogenic by Lee et al 2014 |
| 0202259,  28,  F | [HP:0007108] Demyelinating peripheral neuropathy [HP:0004336] Myelin outfoldings  [HP:0001763] Pes planus | CMT4B2 (604563) | *SBF2* | HMZ  NM_030962.3,  c.620-9T>A | Absent in population databases  Proven to be in trans by segregation analysis in addition unaffected sister HMZ wildtype  Patient’s phenotype specific for gene  Protein truncating effect confirmed on RNA studies |
| 0102007,  65,  M | [HP:0007327] Mixed demyelinating and axonal polyneuropathy | Charlevoix-Saguenay type spastic ataxia, (270550) | *SACS* | HTZ  NM_014363.5, NP_055178.3 c.10906C>T, p.(Arg3636*)  HTZ  NM_014363.5, NP_055178.3 c.8942T>C, p.(Leu2981Pro) | Truncating variant in a gene where loss-of-function mutations are a known disease mechanism.  Predicted to be pathogenic by in-silico analysis  Previously reported as pathogenic by Vermeer et al. 2008  Absent from population databases  Predicted to be pathogenic by in-silico analysis  Located in mutation hotspot  Detected in combination with another pathogenic variant |
| 0102019,  18, M | [HP:0003477]  Axonal neuropathy | Optic atrophy plus syndrome  (125250) | *OPA1* | HMZ  NM_015560.2, NP_056375.2  c.1097G>A, p.(Arg366Gln) | Absent from population databases.  Predicted to be pathogenic by in-silico analysis  Located in functional domain  Phenotype regarded as specific for gene  Proven to be in trans with another variant on segregation studies (unaffected parents and sister all HTZ for variant) |
| 0102018,  68,  F | [HP:0003477]  Axonal neuropathy [HP:0000408]  Progressive sensorineural hearing impairment  [HP:0002073]  Ataxia[HP:0002064]  Spastic gait | Non-syndromic hearing loss | *PDZD7* | HTZ  NM_001195263, NP_001182192.1  c.2107delA, p.(Ser703Valfs*20)  HTZ  NM_001195263, NP_001182192.1  c.1933+1G>A | Truncating variant in a gene where loss of function mutations are a known disease mechanism.  Predicted to be pathogenic by in-silico analysis  Previously reported as pathogenic - Vona et al. 2016  Canonical splice variant in a gene where loss of functions mutations are a known disease mechanism  Absent from population databases  Predicted to be pathogenic by in-silico analysis |
| 0102015,  44,  M | [HP:0003477]  Axonal neuropathy [HP:0002064]  Spastic gait | SPG 31 (610250) | *REEP1* | HTZ  NM_022912.2, NP_075063.1  c.59C>A, p.(Ala20Glu) | Absent from population databases  Predicted to be pathogenic by in-silico analysis  Previously reported as pathogenic by Zuchner et al. 2006 and Beetz et al. 2008, and functional data from Lim et al. 2015 is supportive of it being pathogenic |

Abbreviations: HPO = Human Phenotype Ontology; OMIM = Online Mendelian Inheritance in Man; AD = autosomal dominant; AR = autosomal recessive; XL = X-linked recessive; CMT = Charcot-Marie-Tooth disease; SMA = spinal muscular atrophy; HMN= hereditary motor neuropathy; HSAN-hereditary sensory and autonomic neuropathy; DSD- Dejerine-Sottas disease; HNPP = hereditary neuropathy with liability to pressure palsies. SPG- Spastic Paraplegia

*In-silico prediction tools used Mutationtaster.org (Schwarz JM et al. 2014), Pholyphen at http://genetics.bwh.harvard.edu/pph2/ (Adzhubei IA 2010 et al) and <http://sift.jcvi.org> (Kumar P et al 2009),
