# Supplementary material for: Diagnostic and cost utility of whole exome sequencing in peripheral neuropathy
Source: Ann Clin Transl Neurol. 2017 Apr 26;4(5):318–25. doi: 10.1002/acn3.409 (PMC5420808; doi:10.1002/acn3.409)
Supplement: Supplementary file 3 — Table S5 Summary of Cost Data. [file ACN3-4-318-s003.docx]

**Table S5 - Summary of Cost Data**

| ***Standard pathway*** | Prior costs  (AU$) | Total cost including WES (AU$) | Scenario analysis  (AU$) |
| --- | --- | --- | --- |
| Neurology/neurogenetic appointments | 27848.94 | 27848.94 | 19751.94 |
| Subspecialist appointments | 5972.60 | 5972.60 | 5731.94 |
| Basic biochemistry | 5694.21 | 5694.21 | 5694.21 |
| Complex biochemistry | 16862.90 | 16862.90 | 0.00 |
| Serology/immunology | 580.65 | 580.65 | 580.65 |
| Anatomical pathology | 5041.90 | 5041.90 | 0.00 |
| Imaging (XRs, US, MRI, CT, echocardiogram) | 42684.07 | 42684.07 | 50165.45 |
| Neurophysiology (NCS) | 13437.09 | 13437.09 | 10527.05 |
| Genetics testing (SNP microarray) | 16716.00 | 16716.00 | 16716.00 |
| Genetic testing (other) | 11851.80 | 11851.80 | 11851.80 |
| DNA extraction and shipping | 6070.00 | 6070.00 | 0.00 |
| Operating Theatre/anesthetic costs | 14804.21 | 14804.21 | 0.00 |
| Neurophysiology other | 2947.50 | 2947.50 | 2947.50 |
| Genetic testing (sequencing) | 22114.00 | 22114.00 | 0.00 |
| ***WES diagnostic pathway*** |  |  |  |
| Genetic counsellor appointment | 0.00 | 8830.08 | 8830.08 |
| WES (sequencing, analysis, reporting) | 0.00 | 96000.00 | 96000.00 |
| Genetic counselor review appointment | 0.00 | 7064.16 | 7064.16 |
| **Total cost** | **192625.87** | **304520.11** | **235860.78** |
| Total number of patients | 48 | 48 | 48 |
| Total number of diagnosis made | 0 | 19 | 19 |
| **Average cost per patient** | **4013.04** | **6344.17** | **4913.77** |
| **Average cost per diagnosis** | **N/A** | **16,027.37** | **12,413.73** |
| **Incremental cost per additional diagnosis** | **N/A** | **5889.17** | **2275.52** |

**Abbreviations:** WES=whole exome sequencing**,** XR=X-ray, US=ultrasound, MRI= magnetic resonance imaging, CT= computed tomography, SNP= single nucleotide polymorphism.
