# Supplementary material for: Diagnostic and cost utility of whole exome sequencing in peripheral neuropathy
Source: Ann Clin Transl Neurol. 2017 Apr 26;4(5):318–25. doi: 10.1002/acn3.409 (PMC5420808; doi:10.1002/acn3.409)
Supplement: Supplementary file 4 — Figure S1. Analysis of alternative splicing of SBF2 in patient lymphoblast and fibroblast cell lines. [file ACN3-4-318-s004.pdf]

Figure S-1: Analysis of alternative splicing of *SBF2* in patient lymphoblast and fibroblast cell lines.

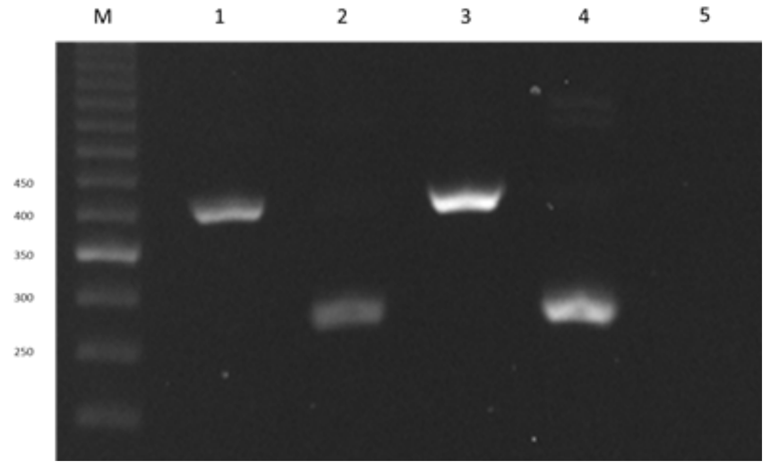

The amplicon products were visualized by agarose gel electrophoresis. Patient samples 2 and 4 showed a reduced sized RT-PCR product (280bp) in comparison to control samples 1 and 3 (399 bp) indicating that the variant caused aberrant mRNA splicing, resulting in the loss of exon 7
